# A trustworthy AI reality-check: the lack of transparency of artificial intelligence products in healthcare

**DOI:** 10.3389/fdgth.2024.1267290

**Published:** 2024-02-20

**Authors:** Jana Fehr, Brian Citro, Rohit Malpani, Christoph Lippert, Vince I. Madai

**Affiliations:** ^1^ Digital Health & Machine Learning, Hasso Plattner Institute, Potsdam, Germany; ^2^ Digital Engineering Faculty, University of Potsdam, Potsdam, Germany; ^3^ QUEST Center for Responsible Research, Berlin Institute of Health (BIH), Charité Universitätsmedizin Berlin, Berlin, Germany; ^4^ Independent Researcher, Chicago, IL, United States; ^5^ Consultant, Paris, France; ^6^ Hasso Plattner Institute for Digital Health at Mount Sinai, Icahn School of Medicine at Mount Sinai, New York, NY, United States; ^7^ Faculty of Computing, Engineering and the Built Environment, School of Computing and Digital Technology, Birmingham City University, Birmingham, United Kingdom

**Keywords:** medical AI, AI ethics, transparency, medical device regulation, trustworthy AI

## Abstract

Trustworthy medical AI requires transparency about the development and testing of underlying algorithms to identify biases and communicate potential risks of harm. Abundant guidance exists on how to achieve transparency for medical AI products, but it is unclear whether publicly available information adequately informs about their risks. To assess this, we retrieved public documentation on the 14 available CE-certified AI-based radiology products of the II b risk category in the EU from vendor websites, scientific publications, and the European EUDAMED database. Using a self-designed survey, we reported on their development, validation, ethical considerations, and deployment caveats, according to trustworthy AI guidelines. We scored each question with either 0, 0.5, or 1, to rate if the required information was “unavailable”, “partially available,” or “fully available.” The transparency of each product was calculated relative to all 55 questions. Transparency scores ranged from 6.4% to 60.9%, with a median of 29.1%. Major transparency gaps included missing documentation on training data, ethical considerations, and limitations for deployment. Ethical aspects like consent, safety monitoring, and GDPR-compliance were rarely documented. Furthermore, deployment caveats for different demographics and medical settings were scarce. In conclusion, public documentation of authorized medical AI products in Europe lacks sufficient public transparency to inform about safety and risks. We call on lawmakers and regulators to establish legally mandated requirements for public and substantive transparency to fulfill the promise of trustworthy AI for health.

## 1 Introduction

Artificial Intelligence (AI) has the potential to reduce burdens and shortages straining overwhelmed healthcare systems (1). Inherent algorithmic biases, however, carry a considerable risk of inflicting harm during deployment (2, 3). AI algorithms learn correlations in the training data and utilize them to make predictions during deployment. When these algorithms are deployed in populations where demographic or clinical characteristics deviate from the training data, previously learned correlations may lead to inaccurate predictions in practice (4, 5). Inaccurate predictions in specific patient groups may propagate health inequities and reproduce racial and gender disparities (6). For example, an algorithm predicting skin melanoma from images of moles may produce inaccurate predictions on dark skin when the training data predominantly contained images of white skin (7).

To ensure a safe translation of AI algorithms into medical practice, it is crucial to understand the design, development, and clinical validation process to infer potential risks of bias and avoiding harm to patients (5, 8–12). Transparency is needed by stakeholders assessing the quality of medical AI software and by their medical end-users and patients. Medical practitioners particularly require transparency, such as evidence about clinical performance and information about safety and risks, because they may be held liable when using AI tools (13, 14). Patients and citizens, on the other hand, require transparency to support their right to know whether the predictions of an AI software are safe and effective for their group (15–18). Although transparency is crucial for evaluating quality, it does not ensure bias-free algorithms. Instead, transparency is necessary to identify and eliminate bias and facilitate continuous improvement and accountability (19). The importance of transparency is reflected in ethical principles for trustworthy AI. The World Health Organization (WHO) (20) and the European Commission's AI High-Level expert group (21) both advocate for public communication on systems’ capabilities, and the development and testing of AI tools. Abundant guidance exists to report on model development (22), training and validation datasets (9, 23, 24), clinical validation and other relevant clinical information (25–28), and facts about performance, safety, and risks stemming from development and test approaches (12, 24, 29–31). Despite the available guidance, experts have raised concerns that principles and guidelines may not be enough to guarantee ethical AI because they lack specific requirements to translate principles into practice (32). An additional challenge is that approaches to measure the compliance with ethical principles currently do not exist (33). Recent research confirmed the challenges to implement trustworthy AI principles in practice, revealing that medical algorithms often pose a high risk of bias and lack transparency about the target population or care setting, prediction target, and handling of missing data (34–37).

An increasing number of AI products are currently available commercially on the European market (38). Yet, it is unclear whether their vendors disclose sufficient information to meet ethical prerequisites for trustworthy AI by adequately informing the public about potential risks. The aim of this paper is to perform a reality-check to determine if approved medical AI tools of a relevant medium to high risk category (Class IIb) fulfil transparency considerations for trustworthy AI. More specifically, we focus on assessing “public transparency”, which we define as ensuring relevant product information is available and accessible to the public.

To conduct this assessment, we applied a survey that we had previously developed and tested to assess the transparency and trustworthiness of medical AI products (30). This survey translates existing guidelines for transparency into specific questions about the (1) intended use, (2) algorithmic development, (3) ethical considerations, (4) technical validation and quality assessment, (5) and caveats for deployment (Supplementary Table S1; Box 1). To gather the necessary data for this assessment, we retrieved publicly available information about all CE-certified medical AI products for radiology with MDR Class IIb listed on the AI for radiology platform (https://grand-challenge.org/aiforradiology/). This information was used to complete our survey. Afterwards, we scored survey responses to introduce a measurable component of transparency that reflects whether the required information was “unavailable” (0 points), “partially available,” (0.5 points) or “fully available” (1 point). Based on these results, we discuss whether publicly available information on CE-marked medical AI products adheres to the ethical considerations of transparency for trustworthy AI (Box 1) (24, 27–29).

**BOX 1 Transparency requirements developed from existing guidelines.**

• *Intended use*: Outline of the intended tasks performed by the AI tool, specification of the predicted output, input data modality, whether use is intended with or without human oversight.
• *Algorithmic development*: Details about the involvement of medical experts during development, the implemented machine learning algorithm, algorithm input and output variables, specification of the training data collection, selection, sources, annotation, preprocessing, and data characteristics.
• *Ethical considerations as per trustworthy AI guidelines*: Risks of potential harm during deployment from erroneous predictions, consent of individuals to provide their data, pseudonymization of data, avoidance of sensitive attributes for prediction making, avoidance of bias, strategies to ensure fairness and monitoring potential biases, human oversight, consultation by ethics review board during development, auditability by third parties, obtained European standard certificates for product safety, general data protection, cybersecurity, and implemented ISO or IEEE standards for data management and governance.
• *Technical validation and quality assessment*: Test data collection, selection, sources, test data characteristics; comparison of the algorithm performance to human medical expert; assessment of fairness, robust performance across multiple settings or devices, explained model predictions (e.g. heatmaps indicating predictive image regions), and cost savings in healthcare by using the AI tool in comparison to traditional processes.
• *Caveats for deployment*: Disclosing in which healthcare settings the product can be used and in which settings or patient groups the applicability has not yet been validated.

## 2 Methods

### 2.1 Data collection

We selected commercially available (CE-marked) medical AI software products from the independent platform “AI for radiology” (38) maintained by the Diagnostic Image Analysis Group from the Radboud university medical center in the Netherlands. This platform was chosen, because it provides the most comprehensive overview of certified AI based software for clinical radiology on the European market. We accessed the product list on January 4th, 2023 and selected available MDR Class IIb medical software products, which are classified as medium to high risk devices as they may influence medical decisions which may cause a serious deterioration of a person's state of health or surgical intervention (39).

For each product, we collected publicly available documentation about the selected software products that was provided by the vendors to the public. The sources for obtaining this documentation included the (1) vendor website, (2) “AI for radiology” platform (38), (3) scientific publications in the ‘Pubmed database’, and the (4) European Database on Medical Devices (EUDAMED).

We browsed vendor websites in a time-sensitive manner (up to 10 min for each vendor) to identify product information, scientific publications, and obtained certificates on compliance to ISO-standards, GDPR or cybersecurity standards.

From the AI for radiology platform, we retrieved the date of market approval and intended use. For one product (Virtual Nodule Clinic by Optellum), no date was listed, and was subsequently obtained from the company's online press release announcing about the CE-marking (https://optellum.com/2022/03/optellum-attains-ce-marking/).

Open access scientific publications were obtained by accessing publication links provided on the AI for radiology platform, the company website, and by searching the PubMed database (https://pubmed.ncbi.nlm.nih.gov/). Scientific publications that did not include co-authors from the vending company were excluded from this assessment to ensure that the obtained information was shared first-hand by the company. Publications that were not open access were not considered publicly available and were therefore excluded from information retrieval.

To obtain product information from EUDAMED, we entered each product name into the “Model/Name” field in the device search engine (https://ec.europa.eu/tools/eudamed/#/screen/search-device) on January 4th, 2023.

### 2.2 Data analysis

We used a previously developed survey-based assessment (30) to assess whether the publicly available product documentation suffices transparency considerations for trustworthy medical AI. The survey was designed to elicit transparent reporting about the model design, development and validation of learning-based AI algorithms that predict health outcomes. The survey includes 78 questions about the (1) intended use of the product, (2) the machine learning methodology (3) training data information (4) implemented ethical considerations, (5) technical and clinical validation conduct and results following medical AI audit proposals (40–42), and (6) caveats for clinical deployment (30). These questions were drawn from existing reporting guidelines for machine learning algorithms (22, 23) in healthcare (9, 27, 43, 44), diagnostic accuracy studies (45), medical AI validation studies (25, 26, 28), and trustworthy AI guidelines (20, 21, 29, 46–48).

We adapted the survey for this study and selected only questions, which we considered relevant for assessing considerations for trustworthy AI according to ethical guidelines (20, 21, 46). (see Box 1). The following changes were additionally applied in comparison to the original questionnaire. First, we reduced questions about the implemented machine learning methodology (Section 2) into one question asking for a summary. Second, we excluded question (Q) 30, “is training data accessible for other researchers or regulatory bodies”, as we did not consider this necessary for trustworthy AI. Third, we excluded Q56 “Was obtained consent revocable”, because we assumed that obtained consent always included the option to revoke. Fourth, we excluded Q61: “Was risk of bias mitigated” because this question may not be applicable if no bias detected. Fifth, we extended the ethics section of this survey with 15 questions on ethical considerations from the Assessment List for Trustworthy AI (ALTAI) provided by the High-Level Expert group for Artificial Intelligence set up by the European Commission (21). The selected ALTAI questions, included questions to reflect strategies for bias oversight and avoidance, human oversight, response mechanisms for adverse effects, cybersecurity certification, data quality monitoring, monitoring of the intended application, implemented GDPR regulations, obtained Standards (ISO, IEEE) for data management and governance, explaining decisions of AI system to user, auditability by third parties and the consultation of an AI ethics review board.

Our final questionnaire for this assessment included 55 questions (Supplementary Table S1). We answered the survey with the obtained public product documentation and the first author JF scored the responses to each question according to the provided degree of transparency on a 3-point scale as either fully disclosed (1 point), partially disclosed (0.5 points) or not disclosed (0 points). Considerations for assigning the scores are listed in Supplementary Table S1. We calculated relative transparency scores across all questions, and each section.

## 3 Results

We identified 14 certified Class IIb medical AI software products from 13 vendors on the AI for radiology platform, which are commercially available on the European market (Table 1). These 14 products were: AI-RAD Companion Prostate MR by Siemens Healthineers (AI-RAD), Annalise Enterprise CXR (Annalise) by Annalise.AI, CAD4TB by Delft Imaging Systems, Koios DS (Koios) by Koios Medical Inc., Oxipit Chest Link (Oxipit) by Oxipit, Quantib Prostate ROI (Quantib) by Quantib, QP Prostate by Quibim, SenseCare Chest DR Pro (SenseCare Chest) and SenseCare Lung Pro (SenseCare Lung), both by SenseTime, Transpara by Screenpoint Medical, Us2.v1 by Us2.ai, Vara by Vara, Veye Lung Nodule (VeyeNodule) by Aidence, and Virtual Nodule Clinic (Virtual Nodule) by Optellum. From here onwards, the abbreviations (indicated in brackets above) of these product names are used.

**Table 1:** Summary of selected products.

	Product name	Vendor	Market entry	Country	Primary intended use of AI	Image modality	# pub	EUDAMED entry
1	AI-RAD Companion Prostate MR	Siemens Healthineers	05-2020	Germany	Prostate segmentation and volume estimation, lesion annotation	Magnetic Resonance	1	no
2	Annalise Enterprise CXR	Annalise.AI	10-2020	Australia	Detection of 124 chest radiography findings for worklist triage	Chest x-ray	3	no
3	CAD4TB	Delft Imaging Systems	10-2014	The Netherlands	Detection of TB-related lung field abnormalities for diagnostic triaging	Chest x-ray	4	no
4	Koios DS	Koios Medical Inc.	12-2021	United States	Lesion/nodule segmentation for breast and thyroid cancer detection	Ultrasound	1	no
5	Oxipit Chest Link	Oxipit	03-2022	Lithuania	Identification of normal chest x-rays, supports 75 different pathologies	Chest x-ray	1	no
6	Quantib Prostate ROI	Quantib	10-2020	The Netherlands	Prostate segmentation for prostate cancer detection	Magnetic Resonance	0	Yes^a^
7	QP Prostate	Quibim	10-2022	Spain	Abnormality detection for prostate cancer detection	Magnetic Resonance	0	no
8	SenseCare Chest DR Pro	SenseTime	04-2021	China	Abnormality detection for worklist order	Chest x-ray	0	Yes^b^
9	SenseCare Lung Pro	SenseTime	10-2020	China	Lung nodule detection and tracking, pneumonia detection	CT	0	no
10	Transpara	Screenpoint Medical	09-2015	The Netherlands	Breast cancer detection aid	Mammography	9	MDD IIa device^c^
11	Us2.v1	Us2.ai	06-2022	Singapore	Detecting heart disease and pulmonary hypertension in transthoracic echocardiograms	Ultrasound	2	no
12	Vara	Vara	10-2019	Germany	Triaging normal exams during breast cancer screening	Mammography	2	no
13	Veye Lung Nodule	Aidence	12-2017	The Netherlands	Lung nodule detection and characterization	CT	1	Yes^d^
14	Virtual Nodule Clinic	Optellum	03-2022	United Kingdom	Lung nodule malignancy prediction of user-selected region	CT	2	no

Listed are the products that were listed as MDR Class IIb on the AI for radiology platform on January 4th, 2023. Product information is given by vendor, market entry, country of the registered company headquarter, the primary intended use by AI, the image modality, the number of available scientific open-access publications (#Pub) and information if the product was listed in the EU-managed database EUDAMED. Links to EUDAMED entries were accessed on January 4th, 2023.

^a^ https://ec.europa.eu/tools/eudamed/#/screen/search-device/ea948bbe-8bc7-46e3-84be-464f4f94ec6c.

^b^ https://ec.europa.eu/tools/eudamed/#/screen/search-device/c79e0e4d-5d1f-44a9-b450-656834f04264.

^c^ https://ec.europa.eu/tools/eudamed/#/screen/search-device/56072790-5200-4f06-a118-746e0b792aaf.

^d^ https://ec.europa.eu/tools/eudamed/#/screen/search-device/6d0cfe24-59bb-47a0-a53a-8123ae9aa7c1.

### 3.1 Obtained information

All vendor websites were available and displayed information about the products (Table 1). We identified scientific publications for ten products (Supplementary Table S2). Three vendors did not publish scientific studies about their products. All other products had between one and nine (average 2.6, median 2.0) open-access publications. Only four products were listed in the EUDAMED database (Quantib Prostate ROI, SenseCare Chest DR, Transpara, and Veye Lung Nodule). Transpara was listed as a MDD Class IIa device, as opposed to the listed MDR Class IIb device on the AI for radiology platform. The other three product entries were listed as MDR Class IIb devices. We found that the listed device information in EUDAMED was scarce and did not include documentation about the design, development, or testing of the device. Only the EUDAMED entry for SenseCare Chest DR informed listed information in the clinical investigation field and informed that no clinical investigation was conducted inside the EU. The other three product entries did not contain information on clinical investigation. The field for ‘Critical warnings or contra-indications’ was filled only for SenseCare Chest DR and stated: “Caution: This product is only used for assisted diagnosis, cannot be used alone for diagnosis. The final diagnosis result should be given by a qualified professional.” The other products lacked information on critical warnings and contra-indications. Obtained quality standard certificates were only listed in the Transpara EUDAMED entry. The other three product entries did not list quality certifications.

### 3.2 Assessment results

We scored the degree of transparency among questions that require relevant documentation for trustworthy AI (Table 2; Supplementary Table S3). We divided the survey into five sections for the five transparency requirements: intended use, algorithmic development, ethical considerations, technical validation and quality assessment, and caveats for deployment. The three products providing the highest transparency were Vara (33.5 points, 60.9%), Annalise (31 points, 56.4%), and US2.v1 (29 points, 52.7%). The four products without scientific publications reached the lowest transparency among all products (SenseCare Lung (3.5 points, 6.4%), SenseCare Chest (4.5 points, 8.2%), and QP Prostate (5 points, 9.1%).

**Table 2:** Excerpt of survey questions and whether information to these questions was available among all 14 products.

Q. Nr.	Question shortform	AI-Rad prostate	Annalise	CAD4TB	Oxipit chest	Koios DS	Quantib	QP prostate	SenseCare chest	SenseCare lung	Transpara	US2.v1	Vara	Veye nodule	Virtual nodule	# Products
1) Intended use																
3	Input data specification	x	x	x	x	x	x	x	x	x	x	x	x	x	x	14
4	Predicted Output specification	x	x	x	x	x	x	x	x	x	x	x	x	x	x	14
2) Algorithmic development																
6	Method summary	x	x	x							x	x	x		x	7
7	Training data locations		x								x	x	x		x	5
9	Time frame of training data collection		x										x		x	3
12	Instruments/Devices										x		x			2
15	Annotation procedure		x									x	x			3
16	Nr. of samples in each class		x								x	x	x		x	5
17	Cross-sectional metadata		x										x		x	3
3) Ethical considerations																
22	Consent		x									x	x		x	4
25	Potential harm				x											1
32	Implemented GDPR	x			x								x			3
37	Monitoring potential biases															0
4) Technical validation and quality assessment																
45	Performance results	x	x	x	x	x					x	x	x	x	x	10
47	Fairness across demographic groups			x							x	x	x		x	5
47	Performance comparison between multiple deployment sites										x	x	x		x	4
48	Performance across clinical outcomes		x		x						x	x	x		x	6
49	Validation of model explanations										x					1
51	Comparison to human expert	x	x	x		x					x	x	x	x		8
5) Caveats for deployment																
53	Demographic groups			x							x		x	x		4
54	Medical contexts		x	x								x	x	x	x	6
55	Additional caveats (i.e. devices)		x	x							x			x		4

Information that was partially or fully available for each product is marked with x. The number of products with available information to these questions is displayed in the last column. Product names were abbreviated for display.

#### 3.2.1 Intended use

**Figure 1:**
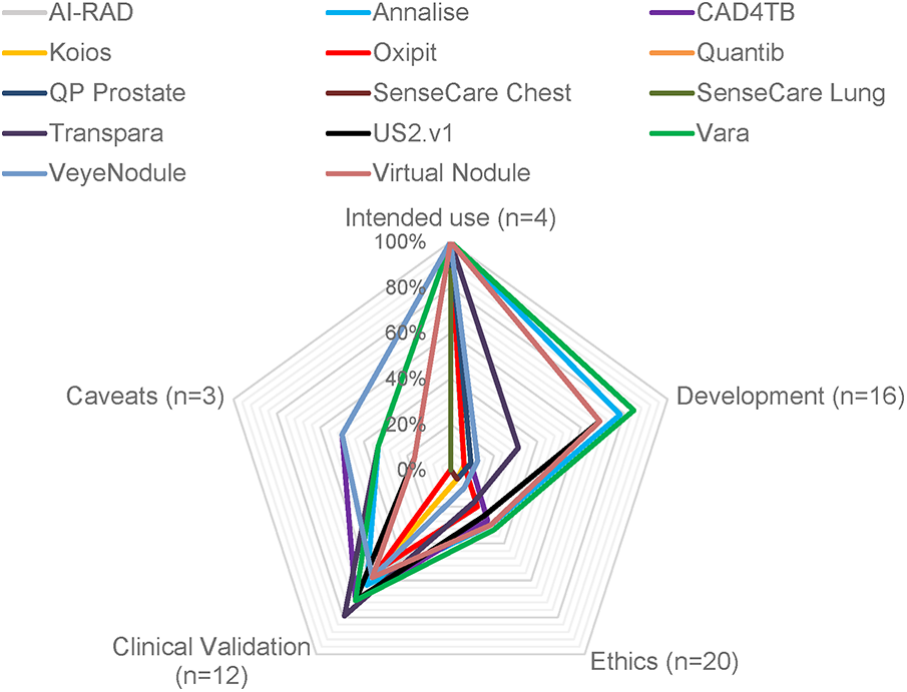
Degree of transparency from publicly available information of 14 CE-certified medical AI tools for radiology. The degree of transparency is grouped by model development, clinical validation results, and ethics. The percentage indicates the transparency of each category relative to the total amount of questions in each category (marked as “*n*=”).

Most products (*n* = 11) provided full information about the intended use (Figure 1). This included information on the medical task performed by the tool, the radiology image modality as input data, and the predicted output by the algorithm. Three products missed to specify the input data or output format. AI-RAD Prostate did not specify necessary magnetic resonance image parameters, such as magnetic field strength or pulse sequences. SenseCare Chest did not specify if the input images include frontal and/or lateral chest x-rays. SenseCare Lung lacked clarity in which format the output of detected pulmonary nodules or pneumonia is presented.

#### 3.2.2 Algorithmic development

Vara (84.4%), Annalise (78.1%), Virtual Nodules (68.8%), and US2.v1 (68.1%) achieved the highest level of transparency on algorithmic development (Figure 1). Ten products provided information on the involvement of clinicians during the development. Seven products gave a methodological summary about the machine learning algorithm used in the device. Five products had documentation on the countries and healthcare settings in which training data was collected. Annalise and Transpara had information on the country but lacked information on the healthcare setting. The remaining nine products had no documentation on training data locations or settings. Three products disclosed the time frame of training data collection, and five products documented selection criteria for training data and sample sizes. Documentation on the radiology devices (vendor and scanner type) that recorded imaging data used for training was only available for two products and only included the vendor, but not the device model. Other cross-sectional demographic and clinical training data characteristics were available for three products (Vara, Annalise, and Virtual Nodules). Three products were transparent about the annotation process used for their training data (Vara, Annalise, and US2.v1). Seven products failed to make any information about the training data publicly available. Missing data handling was described for two products. Three products had information on how the data was preprocessed, and four provided the criteria they used to split datasets into subsets for training and testing.

#### 3.2.3 Ethical considerations

The transparency on ethical considerations achieved by all products on average was 16.6%. Vara (32.5%), Annalise (30.0%), and Virtual Nodule Clinic (30.0%) achieved the highest transparency scores in this section. Four products reported that their training data was de-identified and represented individuals gave consent or an ethics review board waived the need for consent. Documentation on consent and de-identification was missing for the remaining of ten products. Only one product, Oxipit, documented information about potential harm during deployment from misdiagnosis, but deemed this harm neglegible (49). All but one product had information on human oversight, but information on obtained safety certifications and safety-monitoring strategies was limited. We found cybersecurity certificates on two vendor websites, GDPR compliance certifications on three vendor websites, and implemented ISO or IEEE standards on five vendor websites. We identified that six products explain predicted outputs to the end-user. Information about monitoring strategies for data quality, potential biases and fairness were unavailable for all products. No vendor reported if ethical practices were discussed with an AI ethics review board or how the software is auditable by third parties.

#### 3.2.4 Technical validation and quality assessment

Ten products had scientific publications that reported results from the technical validation of the AI model performance in clinical settings. These products achieved scores in the validation section of the survey between 54.2%–79.2%. None of the four products without scientific publications documented clinical validation results on their websites or other sources we considered. Each scientific publication included overall performance results, but the depth of the validation analysis varied between products. The fairness of model predictions across demographic patient groups was partially investigated for five products. For example, Vara published a fairness performance stratification across data from different screening sites and radiology device manufacturers, breast tissue biopsy scores and breast density, but did not investigate performance differences across patient ethnicities. Six products had transparent results from investigating performance differences between different output classes (e.g., pathology subgroups). Five products had documented validation results from multiple deployment sites, but only four stratified the performance across multiple sites. Performance results across multiple imaging device manufacturers was only available for Vara. Transpara was the only product that analyzed and disclosed whether the model explanation output correctly localized the identified pathology. None of the products presented an analysis of the confidence or uncertainty of model predictions. Eight products disclosed the results of a performance comparison between the AI model and human medical expert. Only one product, CAD4TB, shared an analysis on costs saved when using the AI model as compared with traditional medical workflows without use of AI. Two other products (Annalise and Transpara) presented evidence that the reading time of the human experts was reduced when aided by the medical AI software, but we did not consider this as a sufficient cost-efficiency analysis.

#### 3.2.5 Caveats for deployment

Seven products reported caveats for deployment. Three products had limited information on patient subgroups that were underrepresented in the validation data. CAD4TB constrained the use to children above 4 years old, Vara mentioned that elderly women may have been underrepresented, and Transpara mentioned that the training set overrepresented Western populations and that this may explain why the validation performance was lower in an Asian setting. None of the products reported potential performance limitations with respect to multiple demographic characteristics, such as ethnicity and age. The six products that documented the location of training data collection and data selection criteria nonetheless only partially disclosed caveats for deployment. Only two of those products reported underrepresented demographic groups. For example, Vara reported a bias from excluding elderly women due to lacking follow-up data, but did not state a caveat that the software has not yet been tested in settings outside of Germany. The Veye Lung Nodules scientific publication outlined that further investigation is required because the product had only been evaluated at one site and with one scanner device. No product documented a reflection of caveats for all relevant risks of bias during deployment, such as age, gender and ethnicity or country, prevalence-setting, detectable spectrum or stage of pathology and scanner devices.

## 4 Discussion

Public transparency about the use and risks of medical AI algorithms is an essential component of trustworthy AI. Yet, to our knowledge, there is no published investigation examining the extent to which licensed medical AI products on the market implement transparency considerations in practice. In this paper, we addressed this research gap using a survey to systematically investigate whether public information exists for CE-certified MDR Class medium to high risk IIb medical AI tools in radiology on the European market. Our results show that publicly available information for medical AI products on the European market does not meet transparency requirements to inform the public about safety and risks. These findings highlight a gap between the theoretical requirements for trustworthy AI and the reality on the ground. To address this gap, we propose to translate transparency considerations into specific transparency requirements that are legally mandated, enforced by regulatory authorities, and available and accessible to the public.

Our major finding is that the publicly available information of authorized medical AI software does not give sufficient information to inform the public about safety and risks. Most products had no information on training data collection and population characteristics, which is an obstacle to assess the risk of algorithmic bias. Four products had no published results from validation studies. Fairness assessment results across demographic groups were available for only five products. Information on implemented safety monitoring strategies was not publicly shared for any product. Performance limitations were outlined for only half of the products, but none specified deployment constraints for all three potential limiting factors (i.e., demographic groups, clinical settings, or device models). These findings reflect a mismatch between the vast theoretical debate on designing trustworthy AI through transparency and current practices. In practice, vendors have not utilized available reporting frameworks from researchers to provide public transparency about the safety and risks of their medical AI tools (24, 27–29, 50).

So far, the limited documentation of medical AI software has been justified by a lack of understanding on ethics among developers (51–53) or as a threat to intellectual property (IP) (30, 54). Another reason for these identified transparency gaps may be that transparency as a principle for trustworthy AI is only vaguely defined (19, 55). Nonetheless, the primary reason may be that following ethical guidelines is voluntary and not mandated by law. This is especially true for the EU, where the proposed AI Act would require transparency for medical AI products for the first time, but the terms of the law are still under negotiation (56). In the United States, there is no legal obligation to provide product information to the public, but the FDA has released an action plan that calls for “transparency to users about the functioning of AI/ML-based devices to ensure that users understand the benefits, risks, and limitations.” (57).

The European Commission and the FDA both maintain public databases to share information about medical AI products to the public (36, 39). However, listing the devices is not yet legally required, as underlined by the fact that only four of 14 products examined in this study were listed in the EUDAMED database. Although authorities in the United States and Europe encourage companies to disclose product information to the public, there is a lack of specific documentation requirements to uphold the commitment to trustworthy AI. For example, to document the clinical evidence of medical AI products, the EU’s regulation on medical devices requires manufacturers or their sponsors to submit a “clinical investigation report,” a non-technical summary of which later becomes publicly accessible (39). However, there is limited specification what must be made available to the public in the summary report. The EUDAMED database displayed fields related to “Clinical investigation” and “Critical warnings or contra-indications” only for the product SenseCare Chest DR Pro. This information however insufficiently informed about clinical investigation as it listed only that the clinical investigation was conducted in China and a study reference code, but no study details and results. Similarly, another study found that the FDA database also contains scarce documentation about clinical evaluation (58).

Other unspecified requirements are the legal obligations regarding the disclosure of training data information and training data accessibility. Disclosing training data is key to trustworthy AI because it is the “main ingredient” of AI algorithms and a source of bias and safety concerns (8, 24, 59). Researchers and external auditors require access to the training data to conduct quantitative bias assessments and safety checks. Vendors, however, may be unwilling to provide training datasets or summary information owing to aforementioned concerns about IP and trade secrecy (30, 54). Trade secrecy of training data may therefore act as a barrier to public transparency. Currently, both the EUDAMED and the FDA databases seem to support the trade secrecy of vendors because they lack fields to provide information about algorithms or training data. Despite concerns for intellectual property, public transparency, including documentation about the training data and sharing data, is likely to be key to accelerating the adoption of new technology by ensuring safety and reliability (60, 61).

Considering the lack of transparency of medical AI software that our work reveals we call for “public and substantive transparency” for medical AI products: “Public transparency” entails making product information available and accessible to the public, not only to regulatory authorities. “Substantive transparency” means legally mandated, specific, and substantive disclosure requirements, similar to how the term is used other legal contexts (62). Transparency alone does certainly not guarantee bias-free and safe medical AI algorithms, but it is a long-standing requirement for good research practices to enable a subsequent analysis of potential risks (e.g., due to inherent biases) (31, 50). We encourage policy- and decision-makers to draw from existing reporting templates (9, 22–24, 27, 29–31, 50), such as the survey used in this study, to specify and legally mandate transparency requirements for medical AI products. We note that products with peer-reviewed publications achieved higher transparency in our study (22.7%–60.9%) compared to those without publications (6.4%–11.8%). However, scientific publications cannot and should not replace legally mandated public transparency for all products. Since the European public database listing medical AI tools is currently gaining its functionality, we recommend accommodating these mandated transparency requirements as one method to make the information publicly available. This update could also help to keep the workload for vendors manageable by provide transparency in only one database instead of multiple different sites. We also need effective mechanisms to enforce public and substantive transparency requirements in practice. For example, meeting legally mandated transparency requirements could be one component of a pre-market authorization process.

Our method has limitations that could be addressed through future research. First, the reporting survey is an exploratory method to quantify transparency, which requires refinement in future steps. We selected reporting questions based on our subjective interpretation of transparency considerations from trustworthy AI guidelines, but the selection may not cover all relevant considerations for different stakeholders and we did not seek broad-based consensus on the selection. For example, we focused our exploratory analysis on whether vendors report a summary of the implemented algorithm, rather than investigating details such as hyperparameter. Further, we did not include questions on accountability, which is another ethical principle for trustworthy AI (20, 21). Thus, next steps could be to find consensus among multiple stakeholders for selecting reporting questions and developing new approaches to scale or automate transparency assessments. Second, we scored the retrieved information for each question only to the extent if the required information was reported or absent, which might be a source of bias. It is important to note that the scores therefore do not reflect whether the provided information is correct or if the documentation is technically sound. We only analyzed documentation that is provided by the vendors to the public, which may exclude undisclosed information provided to regulatory authorities or to medical customers. Therefore, the results do not represent how transparent the vendors are to their customers or authorities. Also, the selected product information is subject to the timestamp of our analysis and may have already evolved since the retrieval date. Third, it was not possible to identify whether the provided information represents the most recent software version, which has been raised as a general challenge on how to audit medical AI software updates (63). Finally, we had only limited time to conduct transparency audits. Retrieving and reading product information were the most time-consuming tasks. One challenge for this assessment was to conduct the assessment in a feasible timeframe for one auditor (JF). Since we searched for product information in a time-sensitive manner, it cannot be ruled out that more information may be retrieved. Time management also meant that the scope of the study was limited. We would like to point out, however, that this approach likely reflects the reality. It is unlikely that stakeholders would spend hours to find publicly available information. Lastly, we selected only MDR Class IIb products. Our results need to be re-evaluated for other MDR Classes or FDA-approved products.

In summary, we performed for the first time in the literature a reality-check as to whether commercially available medical AI products provide sufficient transparency for trustworthy AI. Our findings highlight major gaps in the documentation on algorithmic development, technical validation and quality assessment, and caveats for deployment. While the regulatory landscape for medical AI is still evolving, we call upon decision-makers to close the gap for implementing ethical guidelines to ensure patient safety and public trust in medical AI (64). In particular, we call for public and substantive transparency—legally mandated specific and substantive transparency requirements for medical AI products that are made available and accessible to the public, not just regulators. We further recommend a participatory process in specifying transparency requirements, recognizing and negotiating the interests of different stakeholders, including patients, health providers, developers, researchers, and regulators.

## Data Availability

The original contributions presented in the study are included in the article/**Supplementary Material**, further inquiries can be directed to the corresponding author.

## Authorȇs note

BC is also a Lecturer at the University of Chicago and RM is a Consultant of Public Health.
