# Supplementary material for: A trustworthy AI reality-check: the lack of transparency of artificial intelligence products in healthcare
Source: Front Digit Health. 2024 Feb 20;6:1267290. doi: 10.3389/fdgth.2024.1267290 (PMC10919164; doi:10.3389/fdgth.2024.1267290)
Supplement: Supplementary file 1 [file Table1.pdf]

Supplementary Material

A Trustworthy AI Reality-Check: The Lack of Transparency of Artificial Intelligence Products in Healthcare.

Jana Fehr[1,2,3], Brian Citro[4], Rohit Malpani[5], Christoph Lippert[1,2,6], Vince I. Madai[3,7]

*** Correspondence:** Vince I. Madai: vince_istvan.madai@bih-charite.de

Contents

Supplementary Table S1: Reporting survey to elicit information about the AI model development and validation, ethical considerations deployment caveats. 1

Supplementary Table S2: Public information for CE-certified MDR class IIb products for radiology. 12

Supplementary Table S3: Transparency assessment results. 16

Supplementary Table S1: Reporting survey to elicit information about the AI model development and validation, ethical considerations deployment caveats.
The survey is structured into six sections on 1) the intended use, 2) implemented machine learning methodology, 3) training data, 4) ethical considerations, 5) technical and clinical validation results, and 6) caveats and recommendations for clinical deployment. Forty questions were selected from [1]. Fifteen questions about ethical considerations were selected from the ALTAI questionnaire [2]. Questions (Q) are numerated, and question numbers in the original survey [1] are indicated. Answers were scored as followed: 0 points if information was not found, 0.5 points if information was incomplete, 1 point if information was fully disclosed. Requirements for scoring 1 point (full information) or 0.5 points (partial information) are indicated.

[1] Fehr, J., Jaramillo-Gutierrez, G., Oala, L., Gröschel, M. I., Bierwirth, M., Balachandran, P., Werneck-Leite, A., & Lippert, C. (2022). Piloting A Survey-Based Assessment of Transparency and Trustworthiness with Three Medical AI Tools. *Healthcare*, *10*(10). https://doi.org/10.3390/healthcare10101923

[2] AI-HLEG (2020). The Assessment List for Trustworthy AI (ALTAI) for self-assessment. https://ec.europa.eu/digital-single-market/en/news/assessment-list-trustworthy-artificial-intelligence-altai-self-assessment

Q#	Q in original survey	Question	Requirement for scoring full information (or partial information if applicable)
		**Section 1 - Intended use**	

1	9	[Multiple answers possible] Specify the primary intended use for the AI model<br>☐ Predicting the onset of a health status change_____________<br>☐ Diagnosing a health problem_______________<br>☐ Predicting health risk__________________<br>☐ Surgery planning ________________________<br>☐ Other ______________________	Specification of the task performed by software
2	10	Should the AI model work autonomously or assistive?<br>☐ Autonomous, to replace health personnel<br>☐ Assistive to support health personnel<br>☐ Other _____________________________	
3	47	[Multiple answers possible] Which data modalities or variables were selected as model input?<br>☐ CT/MRI/X-ray/PET/ images: _________________<br>☐ Microscopy images: ____________________<br>☐ Medical text reports: _____________________<br>☐ Laboratory test results: ___________________<br>☐ Genetic data: ____________________<br>☐ Cognitive test results: ___________________<br>☐ Other __________________________<br>☐ Not disclosed	For imaging data: Settings of image acquisition, e.g. frontal/lateral view X-ray, 1.5T or 3T MRI.
4	15	[Multiple answers possible] Specify the AI model output.<br>☐ Binary classification: The classes are _____________<br>☐ Multiclass (Each sample can only be assigned to one class): The classes are _____________<br>☐ Multilabel (Multiple labels can be assigned to one sample): The labels are ________________<br>☐ Risk score: The scale is__________________<br>☐ Segmented region of interest (ROI). The ROI is... ________________<br>☐ Time until an event occurs. The event is...________________<br>☐ Probability of an event to occur. The event is... ________________<br>☐ Other ____________________	Specification of model output and interpretation, e.g. numeric risk score, final conclusion, or heatmap.
		**Section 2 – Algorithmic development**	
5		Summarize the implemented methodology for the prediction model<br>____	Short summary of implemented machine learning algorithm, including architecture or feature extraction workflow, or citations of implemented methodology.<br><br>Statements of the learning algorithm without details (e.g. deep learning) was considered as insufficient and scored with 0 points.
6	16	Were clinicians consulted during the model development?<br>☐ No	Specification of either no consultation or disclosure in which stages of AI model

		☐ Yes, at the stage(s) ___________________	development clinicans were involved or involvement of medical advisory board or clinical officers.<br>(0.5 points if clinician involvement was indicated, but information on stages or medical advisors unspecified.)
7	29	[Multiple answers possible] Where was the dataset used to develop the model collected?<br>☐ Countries ________________________<br>☐ Cities _________________________<br>☐ Districts (urban/rural)__________________________<br>☐ Health care facilities: __________________________<br>☐ Other: ___________________________<br>☐ Not disclosed	Specification of all: countries, cities, districts (urban/rural), and healthcare facilities (e.g. clinic, screening site, etc.).<br><br>(0.5 points if one of the above points was not reported.)
8	31	[Multiple answers possible] Who collected the dataset? Specify the name of the selected organization<br>☐ A health care facility _______________________<br>☐ An academic institution ___________________<br>☐ A company ____________________<br>☐ A consortium ______________<br>☐ Other ______________________________<br>☐ Not disclosed	
9	34	When was the data collected? Please specify the timeframe.<br>☐ ____<br>☐ Not disclosed	
10	36	How many total data samples does the original dataset contain?<br>☐ less than 100<br>☐ 100–599<br>☐ 600–999<br>☐ 1000–5999<br>☐ 6000–9.999<br>☐ 10.000–99.999<br>☐ 100.000–499.999<br>☐ 500.000–1 Mio<br>☐ More than 1 Mio<br>☐ Not disclosed	
11	45	[Multiple answers possible] How many samples/individuals were selected from the original dataset for developing the model?<br>☐ All samples/individuals from the original dataset were selected for developing the model<br>☐ A subset was selected for model development. Selection criteria and fraction relative to the original dataset were _______________________________<br>☐ Instances/individuals were excluded from model development, if one of the following exclusion criteria were met ________________________<br>☐ Not disclosed	Scored 1 if selected dataset size was not given, but size of original dataset was given under the assumption that all samples in the obtained set were included for model development.
12	38	[Multiple answers possible] Which instruments and settings were used to capture the input data?<br>☐ Camera type and settings ____________________	Specification of device vendor and device model.

		☐ Microscope type and settings _____________________<br>☐ Laboratory assays and tests ________________________<br>☐ Other ________________________<br>☐ Not disclosed	
13	39	If the dataset contained images: Please specify the image size of the original (raw) images.<br>☐ Not applicable<br>☐ All raw images had the image size: ______________________<br>☐ All raw images had varying image sizes in the range of ______________________<br>☐ Information of raw images is not available. The image size of processed available images was ________________<br>☐ Not disclosed	
14	40	Are individuals represented at one or at multiple timepoints in the original dataset? If multiple, please specify time intervals and irregularities.<br>☐ All individuals are represented only at one timepoint<br>☐ Some individuals were recorded only at one timepoint, some at multiple timepoints, depending on ____________________<br>☐ All individuals were recorded at multiple timepoints in regular intervals<br>☐ All individuals were recorded at multiple timepoints in various intervals depending on __________________<br>☐ Other ________________________<br>☐ Not disclosed	
15	41	Are data samples annotated with labels? If yes, how or by whom were these annotated?<br>☐ No label associated with data samples<br>☐ Yes, the labels were annotated by an algorithm<br>☐ Yes, the labels were annotated by X (number) human experts with X (number) years of experience<br>☐ Yes, the labels were obtained from a laboratory test result ____________<br>☐ Yes, the labels were obtained by ____________________<br>☐ Other _____________________<br>☐ Not disclosed	Specification of annotation process and gold standard. If gold standard was derived from a medical test, the test has to be specified. If annotation was done by human experts, a description of number of experts, level of expertise and reading process is required.
16	42	[Multiple answers possible] How many samples of each label class were present in the input dataset?<br>☐ Not applicable<br>☐ Class 1: (class name, % of samples relative to total) ___________<br>☐ Class 2: (class name, % of samples relative to total)<br>☐ Class 3: (class name, % of samples relative to total)<br>☐ Class 4: (class name, % of samples relative to total)<br>☐ Class 5: (class name, % of samples relative to total)<br>☐ More classes: ______________________________<br>☐ Not disclosed	
17	43	[Multiple answers possible] Does the dataset record cross-sectional metadata? Please select present variables and specify the frequencies or appropriate summary statistics.<br>☐ age: ________________________<br>☐ sex: ________________________<br>☐ ethnicity: _______________________	Summary statistics of cross-sectional metadata.

		☐ religion: _________________________<br>☐ type of healthcare visit (routine/emergency): ___________________________<br>☐ stage of disease<br>☐ severity of disease<br>☐ time after first diagnosis<br>☐ time after onset of symptoms<br>☐ time after hospital admission<br>☐ symptoms: _________________________<br>☐ comorbidities: ____________________________<br>☐ Treatment, past or current: __________________<br>☐ Other variables: ____________________________________<br>☐ Not disclosed	
18	44	[Multiple answers possible] How were missing data handled in the original dataset? Specify affected variables or data-modalities, missing fraction relative to all entries and potential reasons for missing data.<br>☐ All data entries were complete<br>☐ The following variables/data modalities were missing. (Missing fractions in %) _________________________<br>☐ Data was missing for unknown reasons<br>☐ Data was missing if/because _______________<br>☐ Other ______________________<br>☐ Not disclosed	Specification of missing data handling required, as we assumed that all datasets contain missing data.
19	48	[Multiple answers possible] Which preprocessing steps were performed to prepare data for ML model development?<br>☐ Resizing/compressing/cropping images to ________________________<br>☐ SIFT feature extraction _____________________<br>☐ Text processing ________________________<br>☐ Missing data imputation using _______________<br>☐ Normalization of image pixel values ____________________<br>☐ Normalization of numeric variables ____________________<br>☐ Other _________________________<br>☐ Not disclosed	
20	50	Did you assign samples to each split at random or stratified by any criteria?<br>☐ At random<br>☐ By matched case-control ____________________<br>☐ Stratification criteria ______________________<br>☐ Not disclosed	
		**Section 3 — Ethical considerations**	
21	54	Were the datasets for developing this model de-identified or anonymized so that individuals cannot be identified?<br>☐ No<br>☐ Yes	

		☐ Other _________________________	
22	55	Did individuals who are represented in this data give consent for using their information for developing this model?<br>☐ Consent was not necessary<br>☐ Oral consent was obtained<br>☐ Written consent was obtained<br>☐ Other ______________________<br>☐ Not disclosed	Specification that either consent was given or consent was waived by ethics review board or lawyers.<br><br>(0.5 points if indication that consent was waived, but no ethics review board or lawyers were involved.)
23	58	[Multiple answers possible] Does the Al model use any sensitive attributes to make predictions? If yes, please specify the attributes.<br>☐ No<br>☐ Ethnicity<br>☐ Sex<br>☐ Religion<br>☐ Age<br>☐ Other ___________________________<br>☐ Not disclosed	Specification of sensitive attributes, or, information that images were the only source of model input.
24	59	Are there any subgroups in which the model might have lower or higher performance compared to others?<br>☐ We do not anticipate that there are subgroups with lower/higher performances, because ______________________________<br>☐ Possibly, but we have not investigated this (yet).<br>☐ We anticipate different performances within the following subgroups _________________________________<br>☐ but we haven't investigated this in detail yet.<br>☐ We found performance differences within the following subgroups _________________________________<br>☐ Other_________________________<br>☐ Not disclosed	Specification of groups where model may potentially have lower performance, because we assume that datasets always underrepresent certain groups.
25	60	[Multiple answers possible] What are potential harms if model predictions are false?<br>Please try to estimate the (1) likelihood that this harm occurs in an application setting and the severity of harm and give reasons for your rating.<br>☐ Not applicable<br>☐ The likelihood is estimated to be low (0–10%) because _________________________<br>☐ The likelihood is estimated to be medium (11–40%) because _________________________<br>☐ The likelihood is estimated to be high (41–100%) because _________________________<br>☐ The severity of potential harm is estimated to be low because _________________________<br>☐ The severity of potential harm is estimated to be medium because _______________________<br>☐ The severity of potential harm is estimated to be high because _________________________<br>☐ Other __________________________<br>☐ Not disclosed	
		ALTAI: Human Agency & Oversight	

26	ALTAI	Please describe whether the AI system (tick as many as appropriate) *<br>Is a self-learning or autonomous system<br>Is overseen by a human-in-the-loop<br>Is overseen by a human-on-the-loop<br>Is overseen by a human-in-command<br>Other<br>Don't know	Clear statement of Human-computer-interaction, or derived information from software information.
27	ALTAI	Have the humans (<u>human-in-the-loop</u>, <u>human-on-the-loop</u>, <u>human-in-command</u>) been given specific training on how to exercise oversight? *<br>Yes<br>No<br>Don't know	Specification of no, or specific offer for training (e.g. online course, customer support)
28	ALTAI	Did you establish any detection and response mechanisms for undesirable adverse effects of the <u>AI system</u> for the <u>end-user</u> or <u>subject</u>? *<br>Yes<br>No<br>Don't know	Specification of yes or no.
		**ALTAI: Technical Robustness and Safety**	
29	ALTAI	Is the AI system certified for cybersecurity (e.g., the certification scheme created by the Cybersecurity Act in Europe) or is it compliant with specific security standards? *<br>Yes<br>No<br>Don't know	Specification of yes or no.
30	ALTAI	Did you put in place measures to ensure that the data (including training data) used to develop the AI system is up to date, of high quality, complete and representative of the environment the system will be deployed in? *<br>Yes<br>No<br>Don't know	Specification of yes or no.
31	ALTAI	Did you put in place a well-defined process to monitor if the AI system is meeting the goals of the intended applications? *<br>Yes<br>No<br>Don't know	Specification of yes or no.
		ALTAI: Privacy and Data Governance	
32	ALTAI	Did you put in place any of the following measures to thoroughly implement the General Data Protection Regulation (GDPR), or non-European equivalent? *<br>Data Protection Impact Assessment (DPIA).<br>Designate a Data Protection Officer (DPO) and include him/her at an early stage in the development, procurement or use phase of the AI system<br>Oversight mechanisms for data processing (incl. limited access by qualified organisation members, mechanisms for logging data access and modifications)<br>Measures to enhance privacy by design and default (e.g., =gb=encryption, pseudonymisation, aggregation, anonymisation=ge=)	Specification of yes or no.

		□ Data minimisation, in particular personal data (incl. special categories of data)	
33	ALTAI	Did you align the AI system with relevant standards (e.g. ISO, IEEE) or widely adopted protocols for (daily) data management and governance? *<br>□ Yes<br>□ No	Specification of yes or no.
		ALTAI: Transparency – Traceability, Explainability & Communication	
34	ALTAI	Did you put in place measures to continuously assess the quality of the input data to the AI system? *<br>□ Yes<br>□ No<br>□ To some extent<br>□ Don’t know	Specification of yes or no.
35	ALTAI	Did you explain the decision of the AI system to the users? *<br>□ Yes<br>□ No<br>□ Don’t know<br>□ Please explain:______________________	Specification of yes or no. Heatmaps or localization markers of predicted findings were considered as explanations.
		ALTAI: Diversity, Non-discrimination and Fairness	
36	ALTAI	Did you establish a strategy or a set of procedures to avoid creating or reinforcing unfair bias in the AI system, both regarding the use of input data as well as for the algorithm design? *<br>□ Yes<br>□ No	Specification of yes or no.
37	ALTAI	Did you assess and put in place processes to test and monitor for potential biases during the entire lifecycle of the AI system (e.g. biases due to possible limitations stemming from the composition of the used data sets (lack of diversity, non-representativeness))? *<br>□ Yes<br>□ No<br>□ To some extent	Specification of yes or no.
38	ALTAI	Did you establish mechanisms to ensure fairness in your AI system? *<br>□ Yes<br>□ No	Specification of yes or no.
		ALTAI: Accountability – Auditability & Risk Management	
39	ALTAI	Did you ensure that the AI system can be audited by independent third parties? *<br>□ Yes<br>□ No<br>□ Don’t know	Specification of yes or no.
40	ALTAI	Did you consider establishing an ‘AI ethics review board’ or a similar mechanism to discuss the overall accountability and ethics practices, including potential unclear grey areas? *<br>□ Yes<br>□ No<br>□ Don’t know	Specification of yes or no.

		**Section 4 —Technical validation and quality assessment**	
41	62	Which type of validation is reported?<br>☐ Internal validation (Split from training data)<br>☐ External validation (data was obtained from an external source which is different from the training data). Please specify how, when and where this evaluation data was collected, and how it's demographics differ from the training data ________________________<br>☐ Not disclosed	Specification of external validation, data source and collection and summary statistics.<br><br>(0.5 points if external validation, but data sources and collection or summary statistics were not available.)
42	63	How many data samples did the validation dataset contain?<br>☐ less than 100<br>☐ 100–599<br>☐ 600–999<br>☐ 1000–5999<br>☐ 6000–9999<br>☐ 10.000–99.999<br>☐ 100.000–499.999<br>☐ 500.000–1 Mio<br>☐ More than 1 Mio<br>☐ Not disclosed	
43	64	[Multiple answers possible] Specify inclusion and exclusion criteria for samples/individuals in the test dataset.<br>☐ All available data for testing was included in the test set<br>☐ The selection was at random<br>☐ The selection was based on the following criteria ___________________________________<br>☐ Samples were excluded from testing if _____________________<br>☐ Not disclosed	
44	65	[Multiple answers possible] How many samples of each label class were present in the test dataset?<br>☐ Class 1: (Name, percent relative to all test samples) ___________<br>☐ Class 2: (Name, percent relative to all test samples) ___________<br>☐ Class 3: (Name, percent relative to all test samples) ___________<br>☐ Class 4: (Name, percent relative to all test samples) ___________<br>☐ Class 5: (Name, percent relative to all test samples) ___________<br>☐ more classes (Name, percent relative to all test samples) __________________________<br>☐ Not applicable<br>☐ Not disclosed	
45	66	[Multiple answers possible] Which performance measures were reported for this evaluation? Please specify the gold standard and respective results.<br>☐ Gold standard: ___________________________<br>☐ Accuracy: ________________________________<br>☐ F1-Score: ________________________________<br>☐ Sensitivity: ________________________________<br>☐ Specificity: ________________________________<br>☐ Precision: ________________________________	Appropriate overall performance measures and results.

		☐ Recall: ________________________________<br>☐ Dice score: ________________________________<br>☐ Area under the curve: ________________________________<br>☐ Area under the precision-recall curve: ________________________________<br>☐ Calibration: ________________________________<br>☐ Other: ________________________________<br>☐ Not disclosed	
46	67	Were plots and tables with evaluation results provided (e.g., ROC-AUC plot)<br>☐ No<br>☐ Yes	Available plots that visualize predictive performance.
47	68	[Multiple answers possible] Was the model performance variations investigated across different groups?<br>☐ No<br>☐ Age groups: _______________________<br>☐ Sex: _______________________<br>☐ Ethnicity: ______________________<br>☐ Deployment sites: __________________<br>☐ Type of healthcare visit (e.g., routine/emergency): __________________________<br>☐ Comorbidity groups: ___________________________<br>☐ Other groups: ___________________________________<br>☐ Not disclosed	Comparison across demographic (age, sex, and ethnicity or diff. countries) groups.
48	69	[Multiple answers possible] Are there clinical output classes or groups (see previous question) for which the Al model performed worse compared to others? (Example early stage of pathology or late stage)<br>☐ The performance was similar for all classes and groups<br>☐ We found performance differences within the following classes/groups (e.g. symptoms, stage of disease) __________________________<br>☐ Not disclosed	Reported comparison across appropriate disease subgroups (e.g. stage of disease and/or symptoms).
49	71	Was a feature-importance analysis reported? (E.g., SHAP or model explanations with e.g. class-activation maps or saliency maps) If yes, which input features were most important, and were they validated for correctness?<br>☐ No<br>☐ Yes, applied method and results were: ____.<br>☐ Not disclosed	Reported feature importance analysis by validating if model always used correct features for predictions (e.g. validating heatmaps by clinicians or validating relative importance per region of interest)
50	72	Was model uncertainty assessed? If yes, which methods and what were the results?<br>☐ No<br>☐ Yes, the analysis approach and results were ____<br>☐ Not disclosed	Reported uncertainty analysis of model predictions, e.g. by calculating and evaluating the confidence of predicted outcomes.<br><br>(0.5 points if indication that confidence scores are calculated, but evaluation results are missing.)
51	73	Was the model performance compared to one or more human experts? If yes, describe the analysis approach, competence level of the human, gold standard and results (e.g., conditions, under which the machine or the human performs better)<br>☐ Not applicable<br>☐ No	Reported comparison to human experts, even if annotations were obtained by human experts.

		☐ Yes, the analysis approach and results were ____<br>☐ Not disclosed	
52	74	Was a cost-efficiency (e.g., saved human hours) analysis conducted to quantify to which extent the model can save healthcare costs? If yes, describe the analysis approach and results.<br>☐ Not applicable<br>☐ No<br>☐ Not yet, but planning to<br>☐ Yes ____<br>☐ Not disclosed	Demonstration of a cost-efficiency analysis comparing medical costs when using the AI tool, vs. without use of AI.
		**Section 5 —Caveats for deployment**<br><br>Are there any limitations to deployment settings where the product may not perform correctly or safely?	
53	76	[Multiple answers possible] Are there relevant demographic subgroups that were not represented or under-represented in the validation dataset and in which Al model performance should be investigated?<br>☐ No, all relevant subgroups were represented in the data and further investigation is not necessary<br>☐ The following subgroups were not/or under-represented in the evaluation data and we recommend further testing for these _________________________________<br>☐ Not disclosed	Specification of demographic subgroups (age, sex, ethnicity or country), which were either underrepresented in the development data, or not included in performance-stratification (fairness) analyses.<br><br>(0.5 points if any of age, sex or ethnicity subgroups were underrepresented or not assessed, but not outlined as caveat.)
54	77	Are there medical settings or clinical groups in which the reported AI tool is not recommended / advisable to be applied?<br>☐ No<br>☐ Yes, ______________________________________<br>☐ Not disclosed	Specification of medical settings (routine screening or healthcare facility and high- or low- burden setting) and clinical subgroups (stage of disease and/or symptoms) that were insufficiently investigated.<br><br>(0.5 points if any of the points were not reported.)
55	78	Additional Caveats (e.g. image acquisition devices)	Specification of caveats against used devices, gold-standards or annotation-procedures.

Supplementary Table S2: Public information for CE-certified MDR class IIb products for radiology. Public sources include open-access publications, product information sheets and information provided on the vendor website.

Product name	Scientific publications	Online information
AI-RAD Companion Prostate MR	• Labus, S., Altmann, M.M., Huisman, H. *et al.* A concurrent, deep learning–based computer-aided detection system for prostate multiparametric MRI: a performance study involving experienced and less-experienced radiologists. *Eur Radiol* **33**, 64–76 (2023). https://doi.org/10.1007/s00330-022-08978-y	Vendor website: https://www.siemens-healthineers.com/<br><br>Product website: https://www.siemens-healthineers.com/digital-health-solutions/ai-rad-companion<br><br>Product Flyer: https://marketing.webassets.siemens-healthineers.com/5a97d3467160570e/bc5987f23c0c/DH_AI-Rad_Companion_Prostate-MR_Flyer.pdf
Annalise Enterprise CXR	• Seah, J. C. Y., Tang, C. H. M., Buchlak, Q. D., Holt, X. G., Wardman, J. B., Aimoldin, A., Esmaili, N., Ahmad, H., Pham, H., Lambert, J. F., Hachey, B., Hogg, S. J. F., Johnston, B. P., Bennett, C., Oakden-Rayner, L., Brotchie, P., & Jones, C. M. (2021). Effect of a comprehensive deep-learning model on the accuracy of chest x-ray interpretation by radiologists: a retrospective, multireader multicase study. *The Lancet Digital Health*, *3*(8), e496–e506. https://doi.org/10.1016/S2589-7500(21)00106-0<br>• Seah, J., Tang, C., Buchlak, Q. D., Milne, M. R., Holt, X., Ahmad, H., Lambert, J., Esmaili, N., Oakden-Rayner, L., Brotchie, P., & Jones, C. M. (2021). Do comprehensive deep learning algorithms suffer from hidden stratification? A retrospective study on pneumothorax detection in chest radiography. *BMJ Open*, *11*, 53024. https://doi.org/10.1136/bmjopen-2021-053024<br>• Gipson, J., Tang, V., Seah, J., Kavnoudias, H., Zia, A., Lee, R., Mitra, B., & Clements, W. (2022). Diagnostic accuracy of a commercially available deep-learning algorithm in supine chest radiographs following trauma. *British Journal of Radiology*, *95*(1134). https://doi.org/10.1259/bjr.20210979	Vendor website: https://annalise.ai/<br><br>Product website: https://annalise.ai/solutions/annalise-cxr/
CAD4TB	• Philipsen, R. H. H. M., Sánchez, C. I., Maduskar, P., Melendez, J., Peters-Bax, L., Peter, J. G., Dawson, R., Theron, G., Dheda, K., & van Ginneken, B. (2015). Automated chest-radiography as a triage for Xpert testing in resource-constrained settings: A prospective study of diagnostic accuracy and costs. *Scientific Reports*, *5*(March), 1–8. https://doi.org/10.1038/srep12215<br>• Melendez, J., Philipsen, R., Chanda-Kapata, P., Sunkutu, V., Kapata, N., & van Ginneken, B. (2017). Automatic versus human reading of chest X-rays in the Zambia National Tuberculosis Prevalence Survey. *International Journal of Tuberculosis & Lung Disease*, *21*(April), 880–886. https://doi.org/10.5588/ijtld.16.0851<br>• Murphy, K., Habib, S. S., Zaidi, S. M. A., Khowaja, S., Khan, A., Melendez, J., Scholten, E. T., Amad, F., Schalekamp, S., Verhagen, M., Philipsen, R. H. H. M., Meijers, A., & van Ginneken, B. (2020). Computer aided detection of tuberculosis on chest radiographs: An evaluation of the CAD4TB v6 system. *Scientific Reports 2020 10:1*, *10*(1), 1–11. https://doi.org/10.1038/s41598-020-62148-y	Vendor website: https://www.delft.care/<br><br>Product website: https://www.delft.care/cad4tb/<br><br>*CAD4TB white paper*. https://thirona.eu/wp-content/uploads/2019/05/CAD4TB_6.0.0_WhitePaper.pdf

	• Koesoemadinata, R. C., Kranzer, K., Livia, R., Susilawati, N., Annisa, J., Soetedjo, N. N. M., Ruslami, R., Philipsen, R., van Ginneken, B., Soetikno, R. D., van Crevel, R., Alisjahbana, B., & Hill, P. C. (2018). Computer-assisted chest radiography reading for tuberculosis screening in people living with diabetes mellitus. *The International Journal of Tuberculosis and Lung Disease*, *22*(9), 1088–1094. https://doi.org/10.5588/ijtld.17.0827	
Koios DS	• Barinov, L., Jairaj, A., Becker, M., Seymour, S., Lee, E., Schram, A., Lane, E., Goldszal, A., Quigley, D., & Paster, L. (2019). Impact of Data Presentation on Physician Performance Utilizing Artificial Intelligence-Based Computer-Aided Diagnosis and Decision Support Systems. *Journal of Digital Imaging*, *32*(3), 408–416. https://doi.org/10.1007/S10278-018-0132-5	Vendor website: https://koiosmedical.com<br><br>Product website: https://koiosmedical.com/products/
Oxipit Chest Link	• Tommi, K.-F., Marko, N., Marianne, H., Naglis, R., Osmo, T., & author, C. (n.d.). *Using Artificial Intelligence to detect chest x-rays with no significant findings in a primary health care setting in Oulu, Finland*. https://arxiv.org/ftp/arxiv/papers/2205/2205.08123.pdf	Vendor website: https://oxipit.ai<br>Product website: https://oxipit.ai/products/chestlink/
Quantib Prostate ROI	Not available	
QP Prostate	Not available	Vendor website: https://quibim.com<br><br>Product website: https://quibim.com/products/qp-prostate/
SenseCare Chest DR Pro	Not available	
SenseCare Lung Pro	Not available	
Transpara	• Kerschke, L., Weigel, S., Rodriguez-Ruiz, A., Karssemeijer, N., & Heindel, W. (2021). Using deep learning to assist readers during the arbitration process: a lesion-based retrospective evaluation of breast cancer screening performance. *European Radiology*, *32*, 842–852. https://doi.org/10.1007/s00330-021-08217-w<br>• Wanders, A. J. T., Mees, W., Bun, P. A. M., Janssen, N., Rodríguez-Ruiz, A., Dalmış, M. U., Karssemeijer, N., van Gils, C. H., Sechopoulos, I., Mann, R. M., & van Rooden, C. J. (2022). Interval Cancer Detection Using a Neural Network and Breast Density in Women with Negative Screening Mammograms. *Radiology*, *303*(2), 269–275. https://doi.org/10.1148/RADIOL.210832<br>• Romero-Martín, S., Elías-Cabot, E., Raya-Povedano, J. L., Gubern-Mérida, A., Rodríguez-Ruiz, A., & Álvarez-Benito, M. (2022). Stand-Alone Use of Artificial Intelligence for Digital Mammography and Digital Breast Tomosynthesis Screening: A Retrospective Evaluation. *Radiology*, *302*(3), 535–542. https://doi.org/10.1148/radiol.211590<br>• Pinto, M. C., Rodriguez-Ruiz, A., Pedersen, K., Hofvind, S., Wicklein, J., Kappler, S., Mann, R. M., & Sechopoulos, I. (2021). Impact of artificial intelligence decision support using deep learning on breast cancer screening interpretation with single-view wide-angle digital breast tomosynthesis. *Radiology*, *300*(3), 529–536. https://doi.org/10.1148/radiol.2021204432<br>• Raya-Povedano, J. L., Romero-Martín, S., Elías-Cabot, E., Gubern-Mérida, A., Rodríguez-Ruiz, A., & Álvarez-Benito, M. (2021). AI-based Strategies to Reduce Workload in Breast Cancer Screening with Mammography and Tomosynthesis: A Retrospective Evaluation. *Radiology*, *300*(1), 57–65. https://doi.org/10.1148/radiol.2021203555<br>• van Winkel, S. L., Rodríguez-Ruiz, A., Appelman, L., Gubern-Mérida, A., Karssemeijer, N., Teuwen, J., Wanders, A. J. T., Sechopoulos, I., & Mann, R. M. (2021). Impact of	Vendor website: https://screenpoint-medical.com<br><br>Product website: https://screenpoint-medical.com/fusion-ai/<br>•

	artificial intelligence support on accuracy and reading time in breast tomosynthesis image interpretation: a multi-reader multi-case study. *European Radiology*, *31*(11), 8682–8691. https://link.springer.com/article/10.1007/s00330-021-07992-w<br>• Lång, K., Hofvind, S., Rodríguez-Ruiz, A., & Andersson, I. (2021). Can artificial intelligence reduce the interval cancer rate in mammography screening? *European Radiology*, *31*(8), 5940–5947. https://doi.org/10.1007/S00330-021-07686-3<br>• Sasaki, M., Tozaki, M., Rodríguez-Ruiz, A., Yotsumoto, D., Ichiki, Y., Terawaki, A., Oosako, S., Sagara, Y., & Sagara, Y. (2020). Artificial intelligence for breast cancer detection in mammography: experience of use of the ScreenPoint Medical Transpara system in 310 Japanese women. *Breast Cancer*, *27*(4), 642–651. https://doi.org/10.1007/S12282-020-01061-8<br>• Rodriguez-Ruiz, A., Lång, K., Gubern-Merida, A., Broeders, M., Gennaro, G., Clauser, P., Helbich, T. H., Chevalier, M., Tan, T., Mertelmeier, T., Wallis, M. G., Andersson, I., Zackrisson, S., Mann, R. M., & Sechopoulos, I. (2019). Stand-Alone Artificial Intelligence for Breast Cancer Detection in Mammography: Comparison With 101 Radiologists. *Journal of the National Cancer Institute*, *111*(9), 916–922. https://doi.org/10.1093/JNCI/DJY222	
Us.2AI	• Tromp, J., Bauer, D., Claggett, B. L., Frost, M., Iversen, M. B., Prasad, N., Petrie, M. C., Larson, M. G., Ezekowitz, J. A., & Solomon, S. D. (2022). A formal validation of a deep learning-based automated workflow for the interpretation of the echocardiogram. *Nature Communications*, *13*(1). https://doi.org/10.1038/s41467-022-34245-1<br>• Tromp, J., Seekings, P. J., Hung, C. L., Iversen, M. B., Frost, M. J., Ouwerkerk, W., Jiang, Z., Eisenhaber, F., Goh, R. S. M., Zhao, H., Huang, W., Ling, L. H., Sim, D., Cozzone, P., Richards, A. M., Lee, H. K., Solomon, S. D., Lam, C. S. P., & Ezekowitz, J. A. (2022). Automated interpretation of systolic and diastolic function on the echocardiogram: a multicohort study. *The Lancet Digital Health*, *4*(1), e46–e54. https://doi.org/10.1016/S2589-7500(21)00235-1	Vendor website: https://us2.ai/
Vara	• Leibig, C., Brehmer, M., Bunk, S., Byng, D., Pinker, K., & Umutlu, L. (2022). Combining the strengths of radiologists and AI for breast cancer screening: a retrospective analysis. *The Lancet Digital Health*, *4*, e507–e519. https://doi.org/10.1016/S2589-7500(22)00070-X<br>• Byng, D., Strauch, B., Gnas, L., Leibig, C., Stephan, O., Bunk, S., & Hecht, G. (2022). AI-based prevention of interval cancers in a national mammography screening program. *European Journal of Radiology*, *152*, 110321. https://doi.org/10.1016/j.ejrad.2022.110321<br>•	Vendor website: https://www.vara.ai/
Veye Lung Nodules	• Murchison, J. T., Ritchie, G., Senyszak, D., Nijwening, J. H., van Veenendaal, G., Wakkie, J., & van Beek, E. J. R. (2022). Validation of a deep learning computer aided system for CT based lung nodule detection, classification, and growth rate estimation in a routine clinical population. *PLOS ONE*, *17*(5), e0266799. https://doi.org/10.1371/JOURNAL.PONE.0266799	Vendor website: https://www.aidence.com<br><br>Product website: https://www.aidence.com/veye-lung-nodules/
Virtual Nodule Clinic	• Baldwin, D. R., Gustafson, J., Pickup, L., Arteta, C., Novotny, P., Declerck, J., Kadir, T., Figueiras, C., Sterba, A., Exell, A., Potesil, V., Holland, P., Spence, H., Clubley, A., O'Dowd, E., Clark, M., Ashford-Turner, V., Callister, M. E. J., & Gleeson, F. v. (2020). External validation of a convolutional neural network artificial intelligence tool to predict malignancy in pulmonary nodules. *Thorax*, *75*(4), 306–312. https://doi.org/10.1136/thoraxjnl-2019-214104	Vendor website: https://optellum.com/<br><br>Product website: https://optellum.com/lung-cancer-prediction-ai/

	• Massion, P. P., Antic, S., Ather, S., Arteta, C., Brabec, J., Chen, H., Declerck, J., Dufek, D., Hickes, W., Kadir, T., Kunst, J., Landman, B. A., Munden, R. F., Novotny, P., Peschl, H., Pickup, L. C., Santos, C., Smith, G. T., Talwar, A., & Gleeson, F. (2020). Veterans Affairs, and 10 Department of Radiology, Tennessee Valley Healthcare System. *Medical Service*, *2*. https://doi.org/10.1164/rccm.201903-0505OC	

Supplementary Table S3: Transparency assessment results. We retrieved publically available product information from open-access publications and used the available information to answer questions about intended use, design and validation of the medical AI product. The answers were scored with either 0, 0.5, or 1, to indicate whether the required information was either unavailable, partially available or fully available. Depicted are the scores for each question and the summarized transparency scores across all questions for each product.

		Products													
	**Question shortform**	AI-Rad Prostate	Annalise	CAD4TB	Oxipit	Koios	Quantib	QP-Prostate	SenseCare XR	SenseCare CT	Transpara	US2.v1	Vara	Veye Nodule	Virtual Nodule
	**1) Intended use**														
1	Specify intended use	1	1	1	1	1	1	1	1	1	1	1	1	1	1
2	Autonomous or assistive	1	1	1	1	1	1	1	1	1	1	1	1	1	1
3	Input data specification	0.5	1	1	1	1	1	1	0.5	1	1	1	1	1	1
4	Predicted output specification	1	1	1	1	1	1	1	1	0.5	1	1	1	1	1
	**2) Algorithmic development**														
5	Clinican consulting	0	1	0.5	1	1	0	1	0	0	1	1	1	1	1
6	Method summary	1	1	1	0	0	0	0	0	0	1	1	1	0	1
7	Data locations	0	0.5	0	0	0	0	0	0	0	0.5	1	1	0	1
8	Who collected data	0	1	0	0	0	0	0	0	0	0	1	1	0	1
9	Time frame of data collection	0	1	0	0	0	0	0	0	0	0	0	1	0	1
10	Number of samples in dataset	0	1	0	0	0	0	0	0	0	1	1	1	0.5	1
11	Selection criteria for development set	0	1	0	0	0	0	0	0	0	0	1	1	0.5	1
12	Instruments	0	0	0	0	0	0	0	0	0	0.5	0	0.5	0	0
13	Image size original	0	0	0	0	0	0	0	0	0	0	0	0	0	0
14	Multiple timepoints?	0	1	0	0	0	0	0	0	0	0	1	1	0	1
15	Annotation	0	1	0	0	0	0	0	0	0	0	1	1	0	0
16	Samples of each label class	0	1	0	0	0	0	0	0	0	1	1	1	0	1
17	Cross-sectional metadata	0	1	0	0	0	0	0	0	0	0	0	1	0	1
18	Missing data	0	0.5	0	0	0	0	0	0	0	0	0	1	0	0
19	Preprocessing	0	0.5	0	0	0	0	0.5	0	0	0	1	0	0	0
20	Split criteria	0	1	0	0	0	0	0	0	0	0	1	1	0	1

3) Ethical considerations and requirements

		AI-Rad Prostate	Annalise	CAD4TB	Oxipit	Koios	Quantib	QP-Prostate	SenseCare XR	SenseCare CT	Transpara	US2. v1	Vara	Veye Nodule	Virtual Nodule
21	Datset de-identified?	0	1	0	0	0	0	0	0	0	0	1	1	0	1
22	Consent	0	1	0	0	0	0	0	0	0	0	1	1	0	1
23	Sensitive attributes	0	1	1	0	0	0	0	0	0	0	1	1	0	1
24	Subgroups with different performance? (hypothetical)	0	0	0.5	0	0	0	0	0	0	0.5	0	0.5	0	0
25	Potential harm	0	0	0	1	0	0	0	0	0	0	0	0	0	0
26	Human oversight	1	1	1	1	1	1	1	1	0	1	1	1	1	1
27	Oversight training for end-users	0	0	1	0	0	0	0	0	0	1	0	0	0	0
28	Response mechanisms for adverse effects	0	0	0	0	0	0	0	0	0	0	0	0	0	0
29	Cybersecurity certificate	1	0	0	0	0	0	0	0	0	0	0	0	0	1
30	Data up to date and high quality	0	0	0	0	0	0	0	0	0	0	0	0	0	0
31	Monitoring intended application	0	0	0	0	0	0	0	0	0	0	0	0	0	0
32	Implemented GDPR	1	0	0	1	0	0	0	0	0	0	0	1	0	0
33	Standard (ISO, IEEE) for data management and governance	1	1	1	0	0	0	0	0	0	0	0	0	1	1
34	Continously assess quality of input data	0	0	0	0	0	0	0	0	0	0	0	0	0	0
35	Explain decisions of AI system to user	0	1	1	1	0	0	0	0	0	1	1	1	0	0
36	Strategy for avoiding unfair bias	0	0	0	0	0	0	0	0	0	0	0	0	0	0
37	Test and Monitor potential biases during lifecycle	0	0	0	0	0	0	0	0	0	0	0	0	0	0
38	Mechanisms to ensure fairness in AI system	0	0	0	0	0	0	0	0	0	0	0	0	0	0
39	Auditable by third parties	0	0	0	0	0	0	0	0	0	0	0	0	0	0
40	AI ethics review board	0	0	0	0	0	0	0	0	0	0	0	0	0	0

4) Technical and clinical validation

		AI-Rad Prostate	Annalise	CAD4TB	Oxipit	Koios	Quantib	QP-Prostate	SenseCare XR	SenseCare CT	Transpara	US2.v1	Vara	Veye Nodule	Virtual Nodule
41	Type of validation	1	1	1	1	1	0	0	0	0	1	1	1	1	1
42	Number of test samples	1	1	1	1	1	0	0	0	0	1	1	1	1	1
43	Inclusion/Exclusion criteria	1	1	1	1	0	0	0	0	0	1	1	1	1	1
44	Number of samples in each label class	1	1	1	1	1	0	0	0	0	1	1	1	1	1
45	Performance measures	1	1	1	1	1	0	0	0	0	1	1	1	1	1
46	Plots provided?	1	1	1	1	1	0	0	0	0	1	1	1	1	1
47	Performance stratification across multiple deployment sites	0	0	0.5	0	0	0	0	0	0	0.5	0.5	0.5	0	0.5
48	Fairness assessment across outcome subgroups	0	0.5	0	0.5	0	0	0	0	0	1	1	1	0	0.5
49	Validation of model explanations	0	0	0	0	0	0	0	0	0	1	0	0	0	0
50	Uncertainty analysis	0	0	0	0	0.5	0	0	0	0	0	0	0	0	0
51	Comparison to human expert	1	1	1	0	1	0	0	0	0	1	1	1	1	0
52	Cost-efficiency analysis	0	0	1	0	0	0	0	0	0	0	0	0	0	0
	**5) Caveats for deployment**														
53	Demographic groups	0	0	0.5	0	0	0	0	0	0	0.5	0	0.5	0.5	0
54	Medical contexts	0	0.5	0.5	0	0	0	0	0	0	0	0.5	0.5	0.5	0.5
55	Additional caveats (i.e. devices, and other)	0	0.5	0.5	0	0	0	0	0	0	0.5	0	0	0.5	0
	**SUM**	15.5	31	21	15.5	12.5	5	6.5	4.5	3.5	23	29	33.5	16.5	28.5
	**Percentage**	28.2%	56.4%	38.2%	28.2%	22.7%	9.1%	11.8%	8.2%	6.4%	41.8%	52.7%	60.9%	30.0%	51.8%
